# Supplementary material for: Predictors of misconceptions, knowledge, attitudes, and practices of COVID-19 pandemic among a sample of Saudi population
Source: PLoS One. 2020 Dec 9;15(12):e0243526. doi: 10.1371/journal.pone.0243526 (PMC7725365; doi:10.1371/journal.pone.0243526)
Supplement: S1 Table — (DOCX) [file pone.0243526.s001.docx]

**S1 Table. Study participants’ knowledge, attitudes, and practices regarding COVID-19 pandemic.**

| **Statements** | **Frequency** | **Percentage** |
| --- | --- | --- |
| **Knowledge** | **Correct Answer** | |
| COVID-19 infection is caused by SARS-CoV-2. | 446 | 23.5% |
| COVID-19 infection is spread via respiratory droplets of the infected person. | 1641 | 86.5% |
| All community members are equally at risk for COVID-19. | 1111 | 58.6% |
| The best way of preventing spread of COVID-19 is social distancing | 1811 | 95.5% |
| The best way of preventing spread of COVID-19 is taking treatment | 1099 | 57.9% |
| Any type of group activity may spread this infection | 1785 | 94.1% |
| A symptomless COVID-19 patient (during incubation period) can’t transmit infection | 1261 | 66.5% |
| The risk of getting infected when travelling by plane is higher | 1247 | 65.7% |
| This virus infection can be avoided by frequent hand washings by soap | 1705 | 89.9% |
| Advising Quarantine to passengers coming from infected areas is a good practice to avoid spread of infection | 1840 | 97.0% |
| Lockdown all over the country will control the spread of this virus | 1742 | 91.8% |
| Closing teaching institutions and shopping malls are effective ways of social distancing | 1825 | 96.2% |
| The most common cause of spread of this infection in any country is traveler from infected area | 1626 | 85.7% |
| Isolation period for infected people and those exposed to infection is 14 days | 1589 | 83.8% |
| **Attitude Questions** | **True Answer** | |
| I am sure that COVID-19 infection will be overcome soon. | 1126 | 56.1% |
| We can overcome this problem by taking precautionary steps | 1878 | 93.6% |
| I understand that this infection is highly contagious | 1906 | 95.0% |
| It is my social responsibility to take safety measures in controlling spread of this infection. | 1957 | 97.6% |
| **Practice questions** | **Yes** | |
| I am avoiding meeting my friends and relatives | 1784 | 89.9% |
| I am avoiding visiting crowded place | 1887 | 95.1% |
| I am avoiding using ATM machine. | 1028 | 51.8% |
| I prefer to walk by stairs then using lift | 1212 | 61.1% |
| I am using face mask outside the home | 1096 | 55.2% |
| I am using soap frequently for handwashing | 1806 | 91.0% |
