# Supplementary material for: Predictors of misconceptions, knowledge, attitudes, and practices of COVID-19 pandemic among a sample of Saudi population
Source: PLoS One. 2020 Dec 9;15(12):e0243526. doi: 10.1371/journal.pone.0243526 (PMC7725365; doi:10.1371/journal.pone.0243526)
Supplement: S2 Table — (DOCX) [file pone.0243526.s002.docx]

**S2 Table. Study participants’ misconceptions and impact of COVID-19 pandemic.**

| **Statements** | **Correct answer** | |
| --- | --- | --- |
|  | **n** | **%** |
| An effective treatment is available against COVID-19. | 842 | 44.4% |
| This virus outbreak is only for short period. | 388 | 20.5% |
| COVID-19 can be spread through frozen food | 606 | 31.9% |
| If a person’s corona test is negative it means he is free from this virus | 318 | 16.8% |
| The outbreak of COVID-19 will be reduced during summer | 338 | 17.8% |
| Eating garlic can prevent coronavirus infection | 828 | 43.6% |
| Females are more vulnerable to develop this infection | 1067 | 56.2% |
| Everyone should be tested for COVID-19 | 720 | 38.0% |
| Only older adults and younger people are at risk | 535 | 28.2% |
| Cats and dogs spread coronavirus | 916 | 48.3% |
| Disposable face masks protect against coronavirus | 621 | 32.7% |
| Hand dryers kill coronavirus | 856 | 45.1% |
| You have to be with someone for 10 minutes to catch the virus | 1018 | 53.7% |
| Rinsing the nose with saline protects against coronavirus | 899 | 47.4% |
| Thermal scanners can diagnose coronavirus | 645 | 34.0% |
| Sipping water every 15 minutes can protect corona infection | 825 | 43.5% |
| The coronavirus will die off when temperature rise in the spring | 551 | 29.0% |
| Coronavirus is the deadliest virus known to man | 920 | 48.5% |
| Flu and pneumonia vaccines protect against COVID-19 | 966 | 50.9% |
| **Impact questions** | **True** | |
| I am very much scared of COVID-19 infection. | 925 | 46.3% |
| I am scared of food shortage during lockdown | 357 | 17.8% |
| COVID-19 is affecting my social, mental and psychological well beings. | 637 | 31.7% |
| The overall impact of this pandemic on me.  (participants were allowed to tick one or more options) |  |  |
| I have become careless | 106 | 5.3% |
| I have realized importance of life | 1784 | 86.2% |
| I have become more religious | 738 | 35.7% |
| The overall impact of this pandemic will be on: (participants were allowed to tick one or more options) | | |
| Healthcare system | 1417 | 68.5% |
| Economic condition of the country | 1583 | 76.5% |
| Economic condition of the people | 1284 | 63.8% |
