# Supplementary material for: Predictors of misconceptions, knowledge, attitudes, and practices of COVID-19 pandemic among a sample of Saudi population
Source: PLoS One. 2020 Dec 9;15(12):e0243526. doi: 10.1371/journal.pone.0243526 (PMC7725365; doi:10.1371/journal.pone.0243526)
Supplement: Attachment — Submitted filename: Response to Reviewers comments.docx [file pone.0243526.s004.docx]

Dear Editor,

We are thankful to the reviewers for their comments on our manuscript titled “Predictors of misconceptions, knowledge, attitudes, and practices of COVID-19 pandemic among a sample of Saudi population and its impact: a cross-sectional study” manuscript ID PONE-D-20-16524.
Please find a point by point response to each of the comments in your previous decision letter. We have modified our manuscript, as suggested by the reviewers. All changes have been hi-lighted in the manuscript. I hope that you will reconsider it for the review.

With best regards,

Dr Mukhtiar baig

**Reviewers' comments:**

Reviewer's Responses to Questions

**Comments to the Author**

1. Is the manuscript technically sound, and do the data support the conclusions?

The manuscript must describe a technically sound piece of scientific research with data that supports the conclusions.Experiments must have been conducted rigorously, with appropriate controls, replication, and sample sizes. The conclusions must be drawn appropriately based on the data presented.

Reviewer #1: Partly

Reviewer #2: Yes

Reviewer #3: Yes

Reviewer #4: Yes

Reviewer #5: No

Reviewer #6: Yes

2. Has the statistical analysis been performed appropriately and rigorously?

Reviewer #1: Yes

Reviewer #2: No

Reviewer #3: Yes

Reviewer #4: Yes

Reviewer #5: Yes

Reviewer #6: Yes

3. Have the authors made all data underlying the findings in their manuscript fully available?

The [PLOS Data policy](http://www.plosone.org/static/policies.action#sharing) requires authors to make all data underlying the findings described in their manuscript fully available without restriction, with rare exception (please refer to the Data Availability Statement in the manuscript PDF file). The data should be provided as part of the manuscript or its supporting information, or deposited to a public repository. For example, in addition to summary statistics, the data points behind means, medians and variance measures should be available. If there are restrictions on publicly sharing data—e.g. participant privacy or use of data from a third party—those must be specified.

Reviewer #1: Yes

Reviewer #2: Yes

Reviewer #3: Yes

Reviewer #4: Yes

Reviewer #5: Yes

Reviewer #6: Yes

4. Is the manuscript presented in an intelligible fashion and written in standard English?

PLOS ONE does not copyedit accepted manuscripts, so the language in submitted articles must be clear, correct,and unambiguous. Any typographical or grammatical errors should be corrected at revision, so please note any specific errors here.

Reviewer #1: No

Reviewer #2: No

Reviewer #3: Yes

Reviewer #4: Yes

Reviewer #5: No

Reviewer #6: Yes

5. Review Comments to the Author

Please use the space provided to explain your answers to the questions above. You may also include additional comments for the author, including concerns about dual publication, research ethics, or publication ethics. (Please upload your review as an attachment if it exceeds 20,000 characters)

Reviewer #1: Thank you for the manuscript on a very current issue.
As for each day, the facts about COVID are changing and newer things are evolving. So, regarding the right Knowledge and concepts, we are still in the way to know much more things. Some facts what we knew previously may be currently modified or outdated. Accordingly the attitude and practice needs modification. Will the findings from this study really help in policy making for

prevention of COVID?

Reply:

Thank you for your comments. You have mentioned rightly that Knowledge regarding the SARS-2 is changing rapidly. Some facts that we knew previously may be currently modified or outdated.

Our study points out the Knowledge, attitude, misconceptions, practicing behaviors, and impact on people and country economic conditions from Saudi Arabia.

It seems a good addition to the existing literature.

As you have asked, “Will the findings from this study really help in policy making for

prevention of COVID?” We would say that such types of studies are always helpful to some extend for the policymakers to take necessary steps to initiate campaigns for educating the masses.

Reviewer #2: Dear authors,
I have read your manuscript critically before placing the following comments.

1. Please note that you have two versions of the abstract. One is structured and the other is not.

Reply: Sorry for this mistake. We have corrected this mistake.

2. Your statement in the Introduction: "Saudi Arabia is also severely hit by this deadly virus. Cases have been recorded in almost all the regions with pocketing of cases in multiple cities."
Although seems to be true, requires an official reference to substantiate it.

Reply: Thank you for pointing out this. A reference has been added.

3. Concerns on the reliability and validity:
You have mentioned: "The questionnaire was translated, and back-translated (English-Arabic) by two bilingual experts’ and the questionnaire was modified according to their suggestions. Two senior faculty members validated the questionnaire. A pilot study on 35 people from the general public was done to determine the convenience and comprehension of the questionnaire and its Cronbach's
alpha was .81."
All I could grasp is that the translation was done, face validity was performed, and a Cronbach's value was derived. Can you please elaborate on the test-retest if it was carried out, secondly, was Principal Component Analysis planned, or why not?

This is used to identify the underlying components. These components or factor loadings will tell you what factors your questions measure.

Reply:

Thank you for these comments. We checked the face validity and Cronbach's value. We did not perform test-retest. You are right about the PCA, but we did not perform it. We have cited 40 references in this manuscript, and non-of the authors performed PCA for such types of COVID19 related KAP study.

4. Can you please describe the process of only reaching the residents of one city, namely Jeddah from Internationally used social media platforms like Whatsapp, Facebook, and Twitter.From the total responses of N=2117, you had to exclude some responses due to incomplete data, and your final sample was N = 2006. Were there no respondents from outside of Jeddah? A description of this in the methodology would be appropriate.

Reply:

Jeddah is the second-largest city in Saudi Arabia, and it's a cosmopolitan city. We removed other cities that spread of the pandemic was variable across the cities, and educational status of the people was varied. Our target was to collect at least one city population's perception of this pandemic. We have included this in the methodology, as suggested.
5. You have mentioned: "The practice scores ranged from 0-12 (yes=2 points, sometimes=1 and No= 0) and =>6 scores was considered adequate and <6 was considered not adequate"
Can you please provide a reference to the cut-off value of 6 that you have utilized to categorize the responses into Adequate and Not Adequate.

Reply:

It was an arbitrary value given to the practice score. We considered that if a person had a < 50% practice score, it was considered not adequate.

6. The question to the respondents: "I understand that this infection is highly contagious"
Please justify as why this question is placed in Attitude section and not in the Knowledge section? As mentioned earlier, a PCA could have been an objective justification if it was carried out.

Reply:

Yes, you are right; it can be placed in attitude as well. However, our focus was to find out either they know it is highly contagious or not. So we placed it in Knowledge.

7. Please provide the 95% confidence intervals in your result tables to judge the precision of the findings.

Reply:

In both regression tables, the 95% confidence intervals have been included.

8. What was your analysis plan for multiple regressions?

Reply:

We have computed multiple linear regression analysis. The dependent variable (Knowledge and practice) were considered numeric variables and independent variables (age, education, nationality, job, marital status, etc.) were categorical, so we used multiple linear regression analysis to compute the association.

9. You have carried out Multiple regression prior to completing the all Binary logistic regressionTable 5: Multiple regression analysis showing predictors of Knowledge and practice.
Table 6: Binary logistic regression analysis Association of attitude and misconception
scores with different variables.

Reply:

Actually, it was a multiple linear regression analysis. The dependent variable (Knowledge and practice) were considered numeric variables and independent variables (age, education, nationality, job, marital status, etc.) were categorical, so we used multiple linear regression analysis to compute the association.

The dependent variable (misconception and attitude) were taken as binominal categorical and independent variables (age, education, nationality, job, marital status, etc.) were also categorical, so logistic regression analysis was applied to compute the association.

Overall, please ensure that English editing is carried out.

Reply:

Language editing has been done.

Reviewer #3: Dear Authors,
Thank you for your efforts to increase our understanding of COVID-19.
Below please find my comments:

Page 5, the middle of Paragraph 1: you mentioned “… an online questionnaire was administered” however, you did not mention which tool/platform you used to generate the quesionnaire. Later on page 5 you almost mentioned the same with more details. “This questionnaire was converted to a google document containing” etc. I think this is a repetition that is is not needed. Please join the information in one paragraph.

Reply:

We have joined the information in one paragraph.

Throughout the paper, you mentioned (like on page 14, first line) “educational status was the predictor for good knowledge”. Please change this and similar statements to “ was a predictor for…” because when you use “the” it shows as this is an absolute thing.

Reply:

Thank you for this suggestion. We have modified as suggested.

The same is mentioned on Page 17 (Discussion, Misconception): the word “the” used so often. Please revise.

Reply:

Thank you for this suggestion. We have modified as suggested.

Your paper lacks Table 1, which usually describes the demographics of the participants. This is very important for the reader to understand your sample. The reader should not wait until he/she gets to the very detailed Table 4 to get that information, which also serves a completely different purpose.

Reply:

Thank you for this suggestion. We have included table 1 related to the basic characteristics of the study participants.

In your paper, you differentiated between sources of information as 1) SM and 2) information from government sources. This was evident in Discussion, Page 17 (under Source of Information). My understanding is that social media (Twitter, WhatsApp, Telegram) is heavily used by the Ministry of Health to spread COVID-related information to the public. So when a participant mentioned the use of SM, how did you make a distinction between using reliable and unreliable SM sources?

Reply:

Thank you for this question. Actually, we gave separate options WhatsApp, Twitter, Instagram, and Govt services. So, we thought that if the participants were using WhatsApp, Twitter, and Instagram messages from government services, they would tick govt services.

Page 18: you mentioned “We found that the older age and people doing their businesses were the predictors of misconception”. What do you exactly mean by “ doing their businesses”? What is the definition for that?

Reply:

Thank you for this question. It means businessmen. We have modified this in the manuscript.

Also, the use of "the predictors" is another example for the point I raised earlier. It should be "were predictors of…"

Reply:

We have modified the sentences.

Page 19, like 5 you mentioned “Nobody intends to have COVID-19 infection, which has a moderately high risk of mortality [24]” I found this to be out of context.

Reply:

We have removed the sentence.

Page 19, line 5 from the bottom of the page, you wrote “The knowledge
of a person is transformed into attitude and later on reflected in practice”. This is a very strong statement that needs evidence.

Reply:

The statement has been rephrased, and a reference has been provided.

Page 20, under Attitudes, third paragraph, you wrote “The Saudi nationality was the predictor of participants’ positive attitude”. First, please rephrase to something like “being a Saudi was predictor to having a positive attitude”. Second, since there was no Table 1, I was able find out that non-saudis represent about 17% of the sample. Is this strong enough to draw a conclusion that being a Saudi predicts a positive attitude?

Reply:

Thank you for pointing out this. The sentence has been rephrased in the whole manuscript. Actually, this statistical conclusion was based on % of the positive attitudes in both groups.

Generally, I do believe that you need to revise the English language of the manuscript. Here are just some examples:
- page 8 in the table: COVID “be” spread. The word be is not correct.

Reply:

It has been corrected, and language editing has been done.

- page 10, the paragraph under the table: “Positive score indicates positive attitude, Negative or zero score indicates negative attitude Correct (true) answer = 1 score, Wrong (false) answer = 0 score,not sure= 0 score ≤ 50% score = Poor (1 – 9 score), > 50% score= good (10 – 19 score), High score indication good concept”. I copied the sentence exactly from the manuscript. English needs revision.

Reply:

It has been revised.

- Page 18, line 3 (Discussion): I believe the word “excess” should be “access” .

Reply:

Yes, you are right. It has been corrected.

- Page 18, first line under impact you wrote "The current pandemic of COVID-19 has resulted in drastic effects on the economic horizon, the world over". Please revise.

Reply:

It has been revised.

- Page 19 first line under Knowledge, you wrote “In our study, the Saudi population (95% had good to moderate knowledge).” Please revise. I think there is a problem with the parentheses.

Reply:

It has been revised.

- Page 20, under Attitudes, line 4 of the second paragraph, Please use COVID-19 instead of corona.

Reply:

It has been corrected.

Reply:

Professional editing services have edited the language.

Reviewer #4: The manuscript is well written with updated and thorough citations. The research is relevant to the public health community and the statistical analysis is very meticulous. There are several grammar and spelling errors highlighted in the comments, in addition to a few comments about certain parts of the manuscript.

Reply:

Thank you for your comments. Language editing has been done.

Reviewer #5:

The aim is not focused enough, knowledge attitude and practices of what ? and impact on what? information is vague, various misconceptions ?, conclusion is not clear. the aim, hypothesis and conclusion along with title should be aligned, not in this case ?

Reply:

Thank you for pointing out this. We have modified these points.

- intro, recent data available should be discussed to present the rationale. The rationale is weak. most literature available is not presented.

Reply:

The rationale has been modified, and few more references have been added.

- The results are all presented in lengthy tables, which are difficult to follow, should be combined with illustrations.

Reply:

Two tables have been removed from the manuscript and added as supplementary files, and one more illustration has been added.

- what sample size was required, how was it calculated, what was the response rate, how does it effect the outcomes of the study.

Reply:

The sample size calculation and has been added to the methodology.

The questionnaire was circulated by social media randomly, so we don't have a record of the response rate.

The discussion has a lot of repetition of results mostly. Discussion should be based on scientific literature reported by other or in other studies, and not mostly on the authors opinions. The discussion is very weak. the discussion is too long, should be limited.

Reply:

We have modified the discussion, and a few new references have been added.

English in the writing is poor and needs revision form native English writer.

Reply:

Language editing has been done by professional editing service.

Scientific writing in the manuscript is poor, what was the hypothesis, it appears that the aim and research question is not clear as the findings are subjective, the value of these findings are limited. Already such data is reported in the literature.

Reply:

The findings of such types of studies are always subjective. You are right that such data is already available, but from our country, less data has been published. So it would add in the existing literature about the awareness and misconceptions of the KSA people.

Your comments are also very subjective. Pls check the other reviewer comments. Reviewer #6: “The manuscript is well written, information was presented clearly, and the topic is addressing an important current topic.”

Reviewer #6:

The manuscript is well written, information was presented in a clear way and the topic is addressing an important current topic. However, there are some few comments to be addressed such as including more papers in the discussion section. Also, include a recommendation section in the discussion and include more important findings in the conclusion section.

Reply:

Thank you for your comments. We have included a few more papers in the discussion. A recommendation section has been added, and conclusions have been rewritten.

6. PLOS authors have the option to publish the peer review history of their article ([what does this mean?](https://journals.plos.org/plosone/s/editorial-and-peer-review-process#loc-peer-review-history)). If published, this will include your full peer review and any attached files.


If you choose “no”, your identity will remain anonymous but your review may still be made public.


**Do you want your identity to be public for this peer review?** For information about this choice, including consent withdrawal, please see our [Privacy Policy](https://www.plos.org/privacy-policy).

Reviewer #1: No

Reviewer #2: **Yes:** Dr. Quadri

Reviewer #3: **Yes:** Asim Al-Ansari

Reviewer #4: No

Reviewer #5: No

Reviewer #6: No

[NOTE: If reviewer comments were submitted as an attachment file, they will be attached tothis email and accessible via the submission site. Please log into your account, locate the manuscript record, and check for the action link "View Attachments". If this link does not appear, there are no attachment files.]

Reply:

We have also modified the manuscript as comments mentioned in the attachment file.
