# Supplementary material for: Predictors of misconceptions, knowledge, attitudes, and practices of COVID-19 pandemic among a sample of Saudi population
Source: PLoS One. 2020 Dec 9;15(12):e0243526. doi: 10.1371/journal.pone.0243526 (PMC7725365; doi:10.1371/journal.pone.0243526)
Supplement: Attachment — Submitted filename: Response to reviewers.docx [file pone.0243526.s005.docx]

PONE-D-20-26398
Predictors of misconceptions, Knowledge, attitudes, and practices of COVID-19 pandemic among a sample of Saudi population

Dear editor,

Pls find the revised manuscript. We have incorporated all the suggestions recommended by the reviewers. We are thankful to the reviewers for their constructive and positive comments. We are sure that the quality of the manuscript has been improved after incorporating all the suggestions.

With best regards,

Dr mukhtiar baig

PLOS ONE


Reviewers' comments:

Reviewer's Responses to Questions

**Comments to the Author**

1. Is the manuscript technically sound, and do the data support the conclusions?

The manuscript must describe a technically sound piece of scientific research with data that supports the conclusions. Experiments must have been conducted rigorously, with appropriate controls, replication, and sample sizes. The conclusions must be drawn appropriately based on the data presented.

Reviewer #1: Yes

Reviewer #2: Yes

Reviewer #3: Partly

2. Has the statistical analysis been performed appropriately and rigorously?

Reviewer #1: Yes

Reviewer #2: Yes

Reviewer #3: No

3. Have the authors made all data underlying the findings in their manuscript fully available?

The [PLOS Data policy](http://www.plosone.org/static/policies.action#sharing) requires authors to make all data underlying the findings described in their manuscript fully available without restriction, with rare exception (please refer to the Data Availability Statement in the manuscript PDF file). The data should be provided as part of the manuscript or its supporting information, or deposited to a public repository. For example, in addition to summary statistics, the data points behind means, medians and variance measures should be available. If there are restrictions on publicly sharing data—e.g. participant privacy or use of data from a third party—those must be specified.

Reviewer #1: Yes

Reviewer #2: Yes

Reviewer #3: Yes

4. Is the manuscript presented in an intelligible fashion and written in standard English?

PLOS ONE does not copyedit accepted manuscripts, so the language in submitted articles must be clear, correct, and unambiguous. Any typographical or grammatical errors should be corrected at revision, so please note any specific errors here.

Reviewer #1: Yes

Reviewer #2: Yes

Reviewer #3: No

5. Review Comments to the Author

Please use the space provided to explain your answers to the questions above. You may also include additional comments for the author, including concerns about dual publication, research ethics, or publication ethics. (Please upload your review as an attachment if it exceeds 20,000 characters)

Reviewer #1:

Q. The article is well written and is comprehensive overall. However, there are some minor corrections needed to be addressed.

The first line in the abstract should be 'The study intends to explore'

Reply:

We have modified the sentence as suggested.

Q. In the introduction, line 71, mentioning words like 'severely' 'deadly' makes the sentence look very dramatic. either remove severely or deadly.

Reply:

We have modified the sentences as suggested.

Q. Again in line 82 and 94 words like 'war against COVID-19' and 'battle against COVID-19' is very dramatic. Use of more subtle words is recommended.

Reply:

We modified the sentences as suggested.

Q. Line 240, in the misconceptions part of discussion, it should be 'Limited data is available..', not are.

Reply:

We have modified the sentence as suggested.

Q. Again in line 364, I think the use of the word 'deadly' is not appropriate as COVID-19 has a spectrum of presentations, from being asymptomatic to having pneumonia etc. it does not always cause critical illness.

Reply:

We have modified the sentence as suggested.

Q. Line 368, use COVID-19 patients instead of corona-infected patients.

Reply:

We have modified the sentence as suggested.

Reviewer #2: Comments to the authors

This manuscript focuses on a relevant issue in the current scenario and the research question is novel. Authors have written the objectives of the study clearly. Abstract gives the summary of the manuscript in a concise manner. However, there are few concerns with respect to this manuscript which need to be addressed by the authors.

Q. Abstract
• Line 52 – Authors can mention some of the prevailing myths in the abstract as well.

Reply:

We have incorporated the few myths in line number 40-43, as suggested.

Q. Methodology What were the inclusion/exclusion criteria?

Reply:

Inclusion and exclusion criteria are given in line 124-126 in methodology.

Q. • Line 125 – what score was given to the responses as not sure?

Reply:

One score was awarded for true, and zero for false and not sure.

Q• Did the scores have weightage based on the type of questions?

Reply:

No. All questions had equal weightage.

Q• Line 146 – why did authors recruit 2117 participants while the estimated sample size was 385? In that case, there is no need to mention about sample size estimation, I believe.

Reply:

We have removed the sample size calculation, as suggested.

Q• It would be appropriate to mention the socio-economic status of the participants instead of mentioning education and occupation separately (table 1).

Reply

It's a nice suggestion, but we have used these variables in regression analysis separately, so it is difficult to replace them with socioeconomic status. So we sorry that we are unable to incorporate this suggestion.

Q• The category Misconception could be classified as Present or Absent instead of Good and Poor.

Reply:

Actually, if we will write misconceptions absent or present, then it will give a different impression.

There were several misconceptions present and after calculating their scores, we categorized them as good concepts and poor concepts.

Q. Results
• Presentation of results within the text is not appropriate. For example …A few common misconceptions were “females are more vulnerable to develop this infection 1067(56.2%), instead it is better to mention only the percentages within the brackets as
A few common misconceptions were “females are more vulnerable to develop this infection (56.2%)

Reply:

Thank you for the suggestion. We have incorporated this suggestion and changed the results accordingly.

Q. • The significant predictors could be mentioned in bold within the tables so that it will easy for the readers to understand the associations between the variables.

Reply:

Thank you for the suggestion. We have mentioned the significant predictors values in bold in the table.

Q • How did the authors assess the mental and psychological wellbeing of the participants? This aspect needs to be mentioned clearly in the methodology.

Reply:

We didn’t use any inventory for this. This was the self-reported disturbed social, mental, and psychological wellbeing due to the pandemic. We have mentioned this in the methods.

Discussion

• Line 270-281 mentions only about other study findings. Authors should highlight their findings and compare it with other study findings and discuss the similarities or differences observed.

Reply:

We have modified the discussion as suggested and removed a few sentences, and added a new reference.

Q• Line 281 – Is it participants' reasons or reasons for Knowledge among participants. Meaning of both would be entirely different.

Reply:

The sentence has been modified.

Q. Grammatical errors need to be corrected in many parts of the manuscript.

Reply:

A professional editing service has done the language editing.

Q• Line 300-301 –What is the relevance of this sentence in the given context? It is not clear what the authors are trying to convey.

Reply:

The sentence has been removed.

Reviewer #3: •

The authors have discussed the study findings which are relevant in the current context.
• The authors need to acknowledge that the sample was not representative & the study design employed was cross-sectional. Hence the results need to be reviewed with caution.

Reply:

Thank you for the suggestion. We have incorporated this suggestion in the limitations.

• Overall English language editing needs to be done & grammatical errors need to be corrected.

Reply:

A professional editing service has done the language editing.

Q• Predictors & Impact are not appropriate terms for cross –sectional study design. Need to be corrected.

Reply:

# Multiple Linear Regression and Logistic Regression analyses are considered predictive analysis. Therefore, we have used the term predictive. In our study impact, related questions were self-reflective questions that's why we had used these terminologies. In literature, a few other studies found that they have used word predictive in cross-sectional studies.

Q• Study participants had a proper realization of the impact of this pandemic. – needs to be corrected

Reply:

We have modified the sentence as suggested.

Q• people causing 860,857 mortalities – mortalities is not correct usage – needs to be rectified.

Reply:

We have replaced the mortalities with deaths.

Q• Cases have been recorded in almost all the regions with pocketing of cases in multiple cities – needs to be reworded.

Reply:

We have modified the sentence as suggested.

Q• on pg 10, 82-95 lines in the later part of introduction is repetitive, needs to be condensed & revised.

Reply:

We have removed a few sentences.

Q• Complete the online questionnaire was considered consent for participation in the survey.-reword. Was there a check box available for the participants to check - I consent

Reply:

We are sorry that there was no check box for consent.

Q• pilot study consisting of 35 people from the general public was conducted to determine the convenience and comprehension of the questionnaire, - -reword

Reply:

We have modified the sentence as suggested.

Q• what was the basis of grading the scores as good, inadequate poor? Was it based on the consensus of experts or the authors or based on published literature?

Reply:

It was based on the consensus of the researchers.

Q• The frequency and percentages were computed for different variables. A chi-squared test was used to explore the comparison between different variables. – needs to be more explicit

Reply:

We have modified the sentence as suggested.

Q• Table 1 – class intervals for age group to be kept uniform

Reply:

Thank you for the suggestion. We have modified the age group intervals and these are uniform now.

Q• Define primary school, college etc in educational level categories as footnotes or in the methods section as educational classification varies between countries.

Reply:

We have written a description of SA education system in the methods.

Q• one-third 637 (31.7%) of the study participants had disturbed social, mental, and psychological wellbeing – how was this assessed?

Reply:

We didn’t use any inventory for this. This is the respondents self-reported statement. We have mentioned this in the methods.

Q• It is preferable not to repeat results in tables & text.

Reply:

We have reduced a few results from the text.

Q• Table 2,3,4 titles to be reworded. The table titles need to be stand alone & self explanatory. Preferable to depict total n in the table title.

Reply:

The title has been modified as per suggestion.

Q• Table 3,4 B values not required, 95% CI can be depicted as -0.235, 0.347 in a single column

Reply:

In Table 3, Knowledge and practice are numeric variables and B values indicate the change in knowledge and practice score by one-unit change in dependents variables used in multiple regression model. In Table 4, B values have been removed.

Q• Table 4 – preferable to write it as unadjusted OR

Reply:

In table 4, adjusted OR was given because we used multiple logistic regression model.

Q• The statistically significant values can be highlighted in bold in tables.

Reply:

We have modified as suggested.

Q• Univariate analysis should be followed by multivariate analysis. The tables have described it in

reverse order.

Reply:

Univariate analysis data was not given in tables.

Q• The same dependant variables used in univariate analysis have to be used for multivariate analysis & different variables cannot be used as shown in Table 3 & 4.

Reply:

Table 3 & 4 have no link with each other. In table 3, dependent variables are Knowledge and practice, and both were taken as numeric, while in table 4, dependent variables are attitude and misconception and both were taken as binary variables (categorical variables).

Q• Initial part of the discussion is mainly focussing on use of SM for source of Knowledge on COVID – needs to be condensed. Discussion should compare & contrast the current study findings with available literature

Reply:

We have modified as suggested.

Q• one-third of the study participants had disturbed social, mental, and psychological wellbeing as mentioned in discussion has not been discussed in the results section

Reply:

This has been described in the result section in line number 208-210.

Q• categorization of misconceptions, basis for the classification of misconceptions is important to discuss in results rather than only p value or 95% CI.

Reply:

In the results section, in the legends of figure 2 it has been described.

Q• mental health/illness was not assessed in the present study nor was it part of the objectives – hence it is not wise to put it in discussion & state it in conclusions

Reply:

We have removed most of the statements and references related to mental health.

Q• Our results are similar to several other studies [8,22,23]. An Australian study described the general public's good Knowledge in Australia, but they had knowledge gaps for a few questions [17] – discussion needs to be more explicit.

Reply:

We have explained this point.

Q• knowledge scores and practicing behaviours were not up to the mark in terms of fighting the spread of COVID-19 [34]. –reword the statement

Reply:

We have modified as suggested.

Q• Our study’s high-level positive attitude may be attributed to the excellent campaign conducted by the MOH for the Saudi population’s awareness – high level positive attitude – words may be used in moderation

Reply:

We have modified the sentence as suggested.

Q• Practices: Almost all the participants attained adequate scores regarding adopting protective measures. Our study participants maintained social distancing and avoided meeting with friends and relatives. Most of the participants frequently used soap for handwashing and used face masks outside the home – most of these statements are pertaining to results of the present study – these need to be described in the results section & not in the discussion.

Reply:

We have deleted these sentences from the discussion as suggested.

Q• Overall the results & discussion have to be reworked on. The discussion section needs to be condensed. Most of the results stated in the discussion section needs to be moved to results section.

Reply:

We have modified as suggested. The discussion section has been condensed, and most of the description of the results have been removed from the discussion.

Q• 12,22,26,27,37 citation of references is incomplete & not in standard format

Reply:

Few references have been updated and two are preprints, so we have given their ODI.

Q• Fig 2 – axis & axis title to be provided

Reply:

We have modified the figure as suggested.

Q• Our study results can be used by policymakers to set priorities in information campaigns on COVID-19 by the MOH/public health authorities and the mass media. – is not appropriate as the study is not based on a representative sample & as authors have acknowledged that only internet users were included. Besides, COVID situation being very dynamic, the study recommendations have to be in moderation.

Reply:

We have modified our recommendations, as suggested.

[NOTE: If reviewer comments were submitted as an attachment file, they will be attached to this email and accessible via the submission site. Please log into your account, locate the manuscript record, and check for the action link "View Attachments". If this link does not appear, there are no attachment files.]

While revising your submission, please upload your figure files to the Preflight Analysis and Conversion Engine (PACE) digital diagnostic tool, <https://pacev2.apexcovantage.com/>. PACE helps ensure that figures meet PLOS requirements. To use PACE, you must first register as a user. Registration is free. Then, login and navigate to the UPLOAD tab, where you will find detailed instructions on how to use the tool. If you encounter any issues or have any questions when using PACE, please email PLOS at [figures@plos.org](mailto:figures@plos.org). Please note that Supporting Information files do not need this step.
