# Supplementary material for: Predictors of misconceptions, knowledge, attitudes, and practices of COVID-19 pandemic among a sample of Saudi population
Source: PLoS One. 2020 Dec 9;15(12):e0243526. doi: 10.1371/journal.pone.0243526 (PMC7725365; doi:10.1371/journal.pone.0243526)
Supplement: Attachment — Submitted filename: Response to reviewer.docx [file pone.0243526.s006.docx]

**Comments to the Author**

1. If the authors have adequately addressed your comments raised in a previous round of review and you feel that this manuscript is now acceptable for publication, you may indicate that here to bypass the “Comments to the Author” section, enter your conflict of interest statement in the “Confidential to Editor” section, and submit your "Accept" recommendation.

Reviewer #1: All comments have been addressed

Reviewer #2: All comments have been addressed

Reviewer #3: All comments have been addressed

2. Is the manuscript technically sound, and do the data support the conclusions?

The manuscript must describe a technically sound piece of scientific research with data that supports the conclusions. Experiments must have been conducted rigorously, with appropriate controls, replication, and sample sizes. The conclusions must be drawn appropriately based on the data presented.

Reviewer #1: Yes

Reviewer #2: Yes

Reviewer #3: Yes

3. Has the statistical analysis been performed appropriately and rigorously?

Reviewer #1: Yes

Reviewer #2: Yes

Reviewer #3: Yes

4. Have the authors made all data underlying the findings in their manuscript fully available?

The [PLOS Data policy](http://www.plosone.org/static/policies.action#sharing) requires authors to make all data underlying the findings described in their manuscript fully available without restriction, with rare exception (please refer to the Data Availability Statement in the manuscript PDF file). The data should be provided as part of the manuscript or its supporting information, or deposited to a public repository. For example, in addition to summary statistics, the data points behind means, medians and variance measures should be available. If there are restrictions on publicly sharing data—e.g. participant privacy or use of data from a third party—those must be specified.

Reviewer #1: Yes

Reviewer #2: (No Response)

Reviewer #3: Yes

5. Is the manuscript presented in an intelligible fashion and written in standard English?

PLOS ONE does not copyedit accepted manuscripts, so the language in submitted articles must be clear, correct, and unambiguous. Any typographical or grammatical errors should be corrected at revision, so please note any specific errors here.

Reviewer #1: Yes

Reviewer #2: No

Reviewer #3: Yes

6. Review Comments to the Author

Please use the space provided to explain your answers to the questions above. You may also include additional comments for the author, including concerns about dual publication, research ethics, or publication ethics. (Please upload your review as an attachment if it exceeds 20,000 characters)

Reviewer #1: As suggested previously, the manuscript was very comprehensive except a few minor changes which have been addressed by the authors in this current revision.

Reply:

Thanks

Reviewer #2: Authors have addressed all the queries raised by the reviewers and edited the manuscript accordingly.

Reply:

Thanks

Reviewer #3: The authors have addressed the queries previously raised. Few minor corrections & grammatical errors to be corrected.

Reply:

Several grammatical and punctuation errors have been corrected.

In the section on Statistical analysis. The collected data were analyzed using SPSS-26. – provide the details of the software.

Reply:

Details have been added.

To investigate the comparison between demographic variables, a chi-squared test was used. It should be chi-square test. The test was employed to assess the association between variables – to reword the statement.

Reply:

Corrections have been done as suggested.

Sample size calculation, basis & rationale for the numbers included needs to be described.

Reply:

Sample size calculation and rationale have been added as suggested.

Table 2. Comparison of knowledge, attitude, practice, and misconceptions scores according to age, gender, nationality, education, job and marital status (n=2006).
n mentioned in results & n in title is different.

Yes, you are right. That's why we didn't include the total number of participants in the table's title in the original manuscript. After the suggestion of one of the reviewers, we included the number of participants. We have deleted the number of study subjects from the title of the study to remove the discrepancy. To further clarify, we have included the sentence "Total number of responses is not same in all categories because of few missing responses” below the table. This discrepancy is due to the fact that few people didn't reply to a few questions.

Reword the title & mention it as socio-demographic variables, instead of naming all the variables in the title.

Reply:

Corrections have been done as suggested and socio-demographic variables have replaced all variable names.

n (%) to be used in column header in table 2, so that % symbol need not be used in every cell of the table.

Reply:

Corrections have been done as suggested.

Table 3 & 4 - zero needs to precede the decimal point in the tables for better readability.

Reply:

Corrections have been done as suggested.

Pg 17 lines-60-62
People have been scared and have mental distress, and these factors are understandable because health is a serious matter for most people. The sentence may be deleted

Reply:

The sentence has been deleted as suggested.

Pg 19 – lines 113 -114
Practices - Our study found good practicing behaviour – to be reworded as good practices & not good practicing behaviour

Reply:

The sentence has been reworded as suggested.

Pg 20 - lines 150-152
Conclusion
Overall, our study participants had good knowledge, highly positive attitudes, and excellent practicing behavior; however, several myths were also prevalent. – to moderate the sentences & avoid usage of highly positive, excellent practice etc

Reply:

The sentence has been reworded as suggested.
